# Level of Evidence and Strength of Recommendations in US Medical Society Clinical Practice Guidelines, 2019–2023: A Cross-Sectional Analysis

**DOI:** 10.1007/s11606-025-10088-6

**Published:** 2025-12-22

**Authors:** Omar Qureshi, Joseph S. Ross, Reshma Ramachandran, Anand R. Habib

**Affiliations:** 1 Yale School of Medicine, New Haven, CT USA; 2 Section of General Medicine, Department of Internal Medicine, Yale School of Medicine, New Haven, CT USA; 3 Department of Health Policy and Management, Yale School of Public Health, New Haven, CT USA; 4 Yale Collaboration for Regulatory Rigor, Integrity, and Transparency, Yale School of Medicine, New Haven, CT USA; 5 National Clinician Scholars Program, Yale School of Medicine, New Haven, CT USA; 6 Division of Hospital Medicine, University of California San Francisco, San Francisco, CA USA; 7 University of California San Francisco School of Medicine, San Francisco, CA USA

**Keywords:** evidence-based medicine, clinical guidelines, medical societies

## Abstract

**Background:**

Clinicians rely on medical societies’ clinical practice guidelines to inform evidence-based patient care. Concordance between these guidelines’ strength of recommendation (SOR) ratings and corresponding level of evidence (LOE) ratings is unclear.

**Objective:**

Evaluate concordance between SOR and LOE ratings for guideline recommendations.

**Design:**

Cross-sectional analysis.

**Research Subjects:**

Recommendations from 309 clinical practice guideline documents issued by 23 US-based medical societies from 2019 to 2023.

**Main Measures:**

Recommendation-level variables: SOR and LOE ratings. Society-level variables: methodology for assigning SOR and LOE ratings and society’s specialty type. SOR-LOE ratings were concordant if the LOE rating was at least as strong as the SOR rating, with concordance quantified using modified Cohen’s kappa [*κ*].

**Key Results:**

Among 7582 recommendations, society guideline writing committees rated SOR as strong for 2303 (30%) recommendations, moderate for 1351 (18%), weak for 2028 (27%), and 1900 recommendations (25%) had no strength assigned. Regarding LOE ratings, 768 (10%) recommendations had strong evidence, 1676 (22%) had moderate evidence, 2421 (32%) had weak or very weak evidence, and 2717 (36%) had evidence lacking an LOE rating. Overall, 5088 (67.1%) recommendations had SOR-LOE rating concordance, and overall SOR-LOE rating concordance was moderate (*κ* = 0.67; 95% confidence interval (CI) 0.66–0.68). Strong recommendations, however, had minimal SOR-LOE concordance (*κ* = 0.27; 95% CI 0.25–0.28; *p* < 0.001), with 1690 (73%) having LOE ratings weaker than strong. Recommendations based on a Modified Grading of Recommendations, Assessment, Development, and Evaluation (GRADE) framework (*κ* = 0.33; 95% CI 0.30–0.37, *p* < 0.001), and issued by obstetrics/gynecology (*κ* = 0.46; 95% CI 0.40–0.51, *p* < 0.001) or neurology/psychiatry (*κ* = 0.22; 95% CI 0.18–0.26, *p* < 0.001) societies had significantly lower rating concordance.

**Conclusions:**

Among recently issued CPG recommendations, overall SOR-LOE rating concordance was moderate, although recommendations with strong SOR ratings had minimal rating concordance. CPG authors could provide clearer rationales for strong recommendations when the corresponding evidence does not reflect commensurate certainty.

**Supplementary Information:**

The online version contains supplementary material available at https://doi.org/10.1007/s11606-025-10088-6.

## INTRODUCTION

Clinical practice guidelines (CPGs) are documents developed by medical societies to enhance the quality and consistency of patient care by offering standardized recommendations pertaining to diagnosis, prevention, treatment, and other clinical decisions. With clinicians’ increasing reliance on CPGs to practice evidence-based medicine, the Institute of Medicine (IOM) formalized quality standards for CPGs in 2011, stating that CPGs “should be based on only the most methodologically rigorous evidence available” in order to be trusted.^1–3^ Reflecting these quality standards and after reviewing evidence for a clinical question, guideline committees rate the level of evidence (LOE) based on the corresponding evidence’s quality, consistency, precision, and methodological integrity.^1,4,5^ LOE ratings generally vary from low-quality, weak evidence (e.g., observational studies, expert opinion) to high-quality, strong evidence (e.g., randomized controlled trials, systematic reviews). Once evidence is synthesized and its quality rated, societies formulate recommendations. Societies assign recommendation strengths, ranging from ungraded to strong, reflecting the balance of anticipated risks and benefits for discrete clinical decisions.^2–4^ Standardized methodologies for grading evidence and assigning recommendation strength, such as the Grading of Recommendations, Assessment, Development, and Evaluation (GRADE) framework, have been adopted by the World Health Organization, National Institutes for Health, and numerous medical societies.^5^

The IOM’s 2011 report emphasized the crucial interplay between LOE and strength of recommendation (SOR) ratings within CPGs and expressed that the two ratings should ideally be concordant to promote clinicians’ trust in recommendations.^1^ IOM suggested that most CPGs had significant room for improvement with respect to concordance between SOR and LOE ratings and that many medical societies lacked sufficient transparency regarding how they assigned SOR ratings based on available evidence.^1^ Importantly, the IOM and GRADE framework acknowledged that LOE and SOR ratings may not be concordant in “paradigmatic situations” where lower-quality evidence could appropriately merit recommendations with a stronger SOR than LOE rating.^1,5–7^

While prior studies have examined SOR and LOE ratings among recommendations offered by individual US-based medical societies,^8–11^ few recent studies have examined the extent to which recommendations’ strengths align with LOE ratings within CPG documents across multiple specialty societies. Accordingly, we evaluated concordance between SOR and LOE ratings for a large sample of recommendations contained within recently issued CPG documents from a diverse set of large, US-based medical societies and identified categories of CPG recommendations with higher or lower SOR-LOE concordance. Busy clinicians rely on top-line recommendation statements from CPG documents as time constraints make conducting their own in-depth evidence appraisals infeasible.^12^ As the IOM’s 2011 report suggested, ensuring that CPG documents’ recommendations are informed by appropriate quality of evidence is critical for maintaining clinicians’ trust in CPG documents and supporting their desire to practice evidence-based medicine.

## METHODS

### Study Design and Reporting

We conducted a cross-sectional study and report the study in accordance with the STROBE guidelines.^13^

### Medical Society Selection

Guided by prior research and workforce data,^14,15^ we identified 30 large, US-based medical societies representing most specialties with the largest numbers of practicing US physicians. We obtained societies’ recent membership totals from their websites.

### Clinical Practice Guideline (CPG) Document Selection

We identified and compiled CPG documents authored by these medical societies using the ECRI Guidelines Trust, a free CPG document clearinghouse.^1,16,17^ CPGs included in our study were published between January 1, 2019, and December 31, 2023, and authored by one or more of the 30 medical societies identified. We excluded “appropriate use criteria.” We deduplicated CPG documents jointly developed by more than one society by assigning authorship to the society in whose proprietary journal the CPG document was published (e.g., American Gastroenterological Association for documents published in *Gastroenterology*) or based on the society named as first author.

For feasibility, we examined ≤ 25 CPGs per society. For societies that published > 25 CPG documents from 2019 to 2023, we chronologically ordered their CPGs and randomly selected 25 CPG documents.

### Data Extraction

From each CPG, we identified distinct recommendations and extracted data for recommendation-level, guideline-level, and society-level variables. Recommendation-level variables included level of evidence (LOE) rating, strength of recommendation (SOR) rating, recommendation setting (outpatient/ambulatory, inpatient, or both), recommendation type (diagnostic, therapeutic, or both), and recommendation subtype (medication, procedure, imaging, lab test, history and exam, surgery, counseling/patient education, treatment approach, subspecialty consult/referral, therapy, other, or multiple). Recommendation subtype was designated as “other” if it did not clearly fit within our pre-specified categories (e.g., lifestyle modifications, personal protective equipment, devices, risk scores, standardization of clinical reporting). Recommendation subtype was designated as “multiple” if it involved multiple diagnostic or therapeutic modalities (e.g., procedures contingent on imaging findings). Guideline document-level variables included the guideline’s focus (disease prevention, disease/symptom management, or both) and whether it was pediatrics-focused or was jointly developed by multiple societies. Society-level variables included the evidence-grading methodology used by the authoring medical society (GRADE, modified GRADE, proprietary/in-house) as ascertained from CPG methodology statements and society websites, and the specialty of the authoring medical society (primary care, primarily outpatient medical specialty, surgical specialty, hospital-based specialty, obstetrics/gynecology, or neurology/psychiatry).

Two study team members independently extracted data from 140 (45%) CPG documents randomly selected across societies. Differences in data extraction for these 140 documents were discussed and reconciled. Agreement in data prior to reconciliation was 91.5%. One study team member then extracted data from the remaining 169 CPGs (55%) using the consensus approach.

### Variable Categorization

LOE rating was our primary independent variable. LOE rating was categorized as “strong,” “moderate,” “weak”, “very weak,” or “none.” Since not all medical societies clearly distinguished weak and very weak quality evidence, we combined these categories into a single “weak/very weak” category. Where LOE rating was “insufficient” or unclear, we considered LOE rating as “none.” Our dependent variable was SOR rating. Following medical societies’ conventions, we categorized SOR ratings as “strong,” “moderate,” “weak,” or “none.” “Conditional” recommendations were categorized as “weak.”

### Statistical Analyses

We used descriptive statistics to characterize our cohort of recommendations based on the aforementioned variables. We then determined the degree of concordance between recommendations’ SOR and LOE ratings. We considered SOR and LOE ratings concordant if a recommendation’s LOE rating was at least as strong as its SOR rating. For example, we characterized moderate strength recommendations informed by strong or moderate levels of evidence as concordant; those informed by weak, very weak, or no assigned level of evidence were not concordant.

We used modified Cohen’s kappa (“modified kappa,” *κ*) statistics to assess concordance between SOR and LOE ratings for recommendations using our abovementioned definition of concordance.^18^ Concordance based on kappa statistic values was interpreted as follows: ≤ 0.20 (none), 0.21–0.39 (minimal), 0.40–0.59 (weak), 0.60–0.79 (moderate), 0.80–0.90 (strong), and > 0.90 (almost perfect).^19^

We estimated modified kappa statistics and 95% confidence intervals (CIs; two-sided *α* = 0.05) with bootstrapping (1000 iterations) for the overall cohort of recommendations and within strata of SOR rating (e.g., concordance between SOR and LOE ratings for recommendations rated “strong”). We used recommendation-level, guideline-level, and society-level variables to understand how well concordance between SOR and LOE ratings varied across subgroups. Finally, we conducted a sensitivity analysis examining overall SOR-LOE rating concordance excluding recommendations with no assigned SOR rating, as these recommendations would, by our definition, have 100% concordance with their LOE rating.

Kappa statistic pairwise comparisons using bootstrapping (1000 iterations) with Bonferroni correction identified statistically significant differences in kappa estimates between subgroups, as differences can be statistically significant (or not) regardless of overlap in 95% CIs.^18,20,21^

Analyses used R version 4.2.3 (Vienna, Austria).

### Institutional Review Board (IRB) approval

This study did not involve human subjects research. IRB approval was neither sought nor necessary.

### Role of the Funding Source

No project-specific external funding source supported this study.

## RESULTS

Our initial search of CPG documents identified 581 CPGs issued by 24 of the 30 societies; six societies did not issue CPGs between 2019 and 2023. We excluded 180 “appropriate use criteria” CPGs (including all American College of Radiology documents), 10 duplicated CPGs developed by multiple societies, and 1 CPG without clearly discernible recommendations. Three societies published > 25 CPGs between 2019 and 2023. We randomly sampled 25 from each and excluded their remaining 81 documents. The final cohort consisted of 309 CPG documents from 23 medical societies (Supplement Fig. 1).

**Figure 1:**
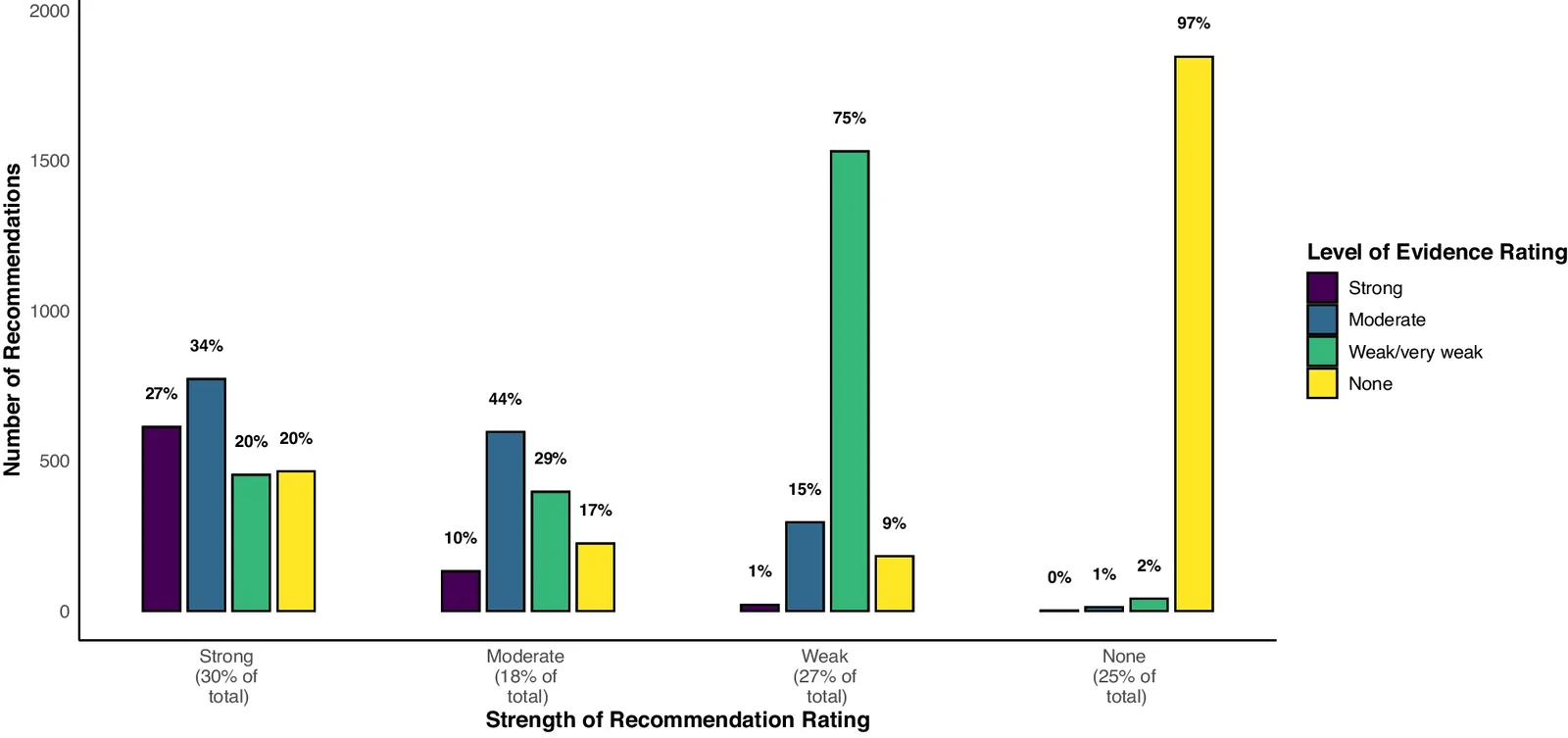
Level of evidence for clinical practice guideline recommendations (*n* = 7582), stratified by strength of recommendation rating.

### Recommendation, Guideline Document, and Society Characteristics

The 309 CPGs contained 7582 recommendations (Supplement Tables 1 and 2). Regarding recommendation-level variables, 5017 (66%) recommendations were related to therapeutic actions or interventions, 4375 (58%) pertained to outpatient/ambulatory care, and 2307 (30%) pertained to medications (Table 1). Regarding guideline document-level variables, 6629 (87%) recommendations focused on disease management, 561 (7%) were pediatrics-focused, and 4080 (54%) were developed jointly by multiple societies. There were 4474 (59%) recommendations authored by primarily outpatient medical subspecialty societies, 1059 (14%) by hospital-based specialty societies, 1060 (14%) by surgical specialty societies, and 286 (4%) by primary care societies. Of the 23 medical societies, 14 (61%) societies used the GRADE framework as their primary method for assigning LOE ratings, 3 (13%) used a modified version of GRADE, and 6 (26%) used a proprietary or in-house evidence-grading framework (Table 2).

**Table 1:** Characteristics of Clinical Practice Guideline Recommendations issued by 23 U.S.-based Medical Societies, 2019-2023

Characteristic		Recommendations (*N* = 7852) No. (%)*
**Recommendation-level**		
	Recommendation setting	
	Inpatient	2029 (27%)
	Outpatient/ambulatory	4375 (58%)
	Both	1178 (16%)
	**Recommendation type**	
	Diagnostic	2293 (30%)
	Therapeutic	5017 (66%)
	Both	272 (4%)
	**Recommendation subtype**	
	Counseling/patient education	301 (4%)
	History and exam	325 (4%)
	Lab test	537 (7%)
	Imaging	376 (5%)
	Medication	2307 (30%)
	Procedure	853 (11%)
	Subspecialty consult/referral	128 (2%)
	Surgery	510 (7%)
	Therapy^†^	87 (1%)
	Treatment approach	826 (11%)
	Other	442 (6%)
	Multiple	890 (12%)
**Guideline document-level**		
	**Guideline document focus**	
	Disease management	6629 (87%)
	Disease prevention	334 (4%)
	Both	619 (8%)
	**Pediatric focus**	
	Yes	561 (7%)
	No	7021 (93%)
	**Guideline document developed jointly by multiple societies**	
	Yes	4080 (54%)
	No	3502 (46%)
**Society-level**		
	**Society evidence grading methodology**	
	GRADE	4254 (56%)
	Modified GRADE	667 (9%)
	Proprietary/in-house methodology	2661 (35%)
	**Society’s specialty categorization**	
	Hospital-based specialty	1059 (14%)
	Medical specialty	4474 (59%)
	Neurology/psychiatry	364 (5%)
	Obstetrics/Gynecology	339 (4%)
	Primary care	286 (4%)
	Surgical specialty	1060 (14%)

^*^Percentages may not sum to 100% due to rounding

^†^The “Therapy” recommendation subtype includes modalities of treatment including physical therapy, occupational therapy, cognitive behavioral therapy, and psychotherapy

**Table 2:** U.S.-based Medical Societies’ Methods of Grading Evidence When Developing Clinical Practice Guideline Documents and Corresponding Number of Guideline Documents and Recommendations Analyzed

Society’s methodological approach to evidence grading	Society name	Medical society specialty category	Most recently reported society membership total	Number of guideline documents selected (% of total)	Number of recommendations (% of total)
**GRADE framework**				205 (66%)	4254 (56%)
	American Academy of Dermatology (AAD)	Medical specialty	20,500	9 (3%)	436 (6%)
	American Academy of Orthopedic Surgeons (AAOS)	Surgical specialty	39,000	17 (6%)	300 (4%)
	American College of Chest Physicians (ACCP)	Medical specialty	22,000	16 (5%)	275 (4%)
	American College of Gastroenterology (ACG)	Medical specialty	16,000	22 (7%)	817 (11%)
	American College of Physicians (ACP)	Primary care	31,000	16 (5%)	63 (1%)
	American Gastroenterological Association (AGA)	Medical specialty	16,000	25 (8%)	293 (4%)
	American Psychiatric Association (APA)	Neurology/Psychiatry	38,900	2 (1%)	40 (1%)
	American Society of Clinical Oncology (ASCO)	Medical specialty	50,000	25 (8%)	472 (6%)
	American Society of Gastrointestinal Endoscopy (ASGE)	Medical specialty	16,000	15 (5%)	107 (1%)
	American Society of Hematology (ASH)	Medical specialty	18,000	15 (5%)	329 (4%)
	American Thoracic Society (ATS)	Medical specialty	16,000	19 (6%)	254 (3%)
	College of American Pathologists (CAP)	Hospital-based specialty	18,000	8 (3%)	112 (1%)
	Society of Critical Care Medicine (SCCM)	Hospital-based specialty	17,000	8 (3%)	341 (4%)
	The Endocrine Society (TES)	Medical specialty	18,000	8 (3%)	415 (5%)
**Modified GRADE framework**				40 (13%)	667 (9%)
	American Academy of Family Physicians (AAFP)	Primary care	130,000	2 (< 1%)	4 (< 1%)
	American Academy of Neurology (AAN)	Neurology/Psychiatry	40,000	13 (4%)	324 (4%)
	American College of Obstetrics and Gynecology (ACOG)	Obstetrics/Gynecology	61,727	25 (8%)	339 (4%)
**Proprietary/in-house grading methodology**				64 (21%)	2661 (35%)
	American Academy of Otolaryngology - Head and Neck Surgery Foundation (AAO HNS)	Surgical specialty	13,000	7 (2%)	107 (1%)
	American Academy of Pediatrics (AAP)	Primary care	67,000	14 (5%)	219 (3%)
	American College of Cardiology (ACC)	Medical specialty	49,000	9 (3%)	1076 (14%)
	American College of Emergency Physicians (ACEP)	Hospital-based specialty	38,000	7 (2%)	47 (1%)
	American Society of Anesthesiologists (ASA)	Hospital-based specialty	57,991	9 (3%)	559 (7%)
	American Urological Association (AUA)	Surgical specialty	25,000	18 (6%)	653 (9%)
**Total**				**309 (100%)**	**7582 (100%)**

### Recommendation Strength, Level of Evidence, and Overall SOR-LOE Concordance

Societies rated 2303 (30%) recommendations as strong, 1351 (18%) as moderate, 2028 (27%) as weak, and 1900 (25%) had no SOR rating (Fig. 1). With regard to LOE ratings, 768 (10%) recommendations were supported by strong evidence, 1676 (22%) by moderate evidence, 2421 (32%) by weak or very weak evidence, and 2717 (36%) by no evidence or evidence for which no LOE rating was assigned.

Overall, 2494 (33%) recommendations had SOR ratings discordant with LOE ratings. Of 2303 strongly rated recommendations, 1690 (73%) were discordant with their corresponding LOE rating, with 772 (34%) informed by moderate evidence, 453 (20%) by weak or very weak evidence, and 465 (20%) by no evidence or evidence with no LOE rating (Fig. 1). Of 1351 moderate strength recommendations, 622 (46%) were discordant with LOE rating, with 397 (29%) informed by weak or very weak evidence and 225 (17%) informed by no evidence or evidence with no LOE rating. Of 2028 weak recommendations, 182 (9%) were discordant with LOE rating as they were informed by no evidence or evidence with no assigned strength. All 2717 recommendations with no SOR rating were considered concordant with their LOE rating.

**Figure 2:**
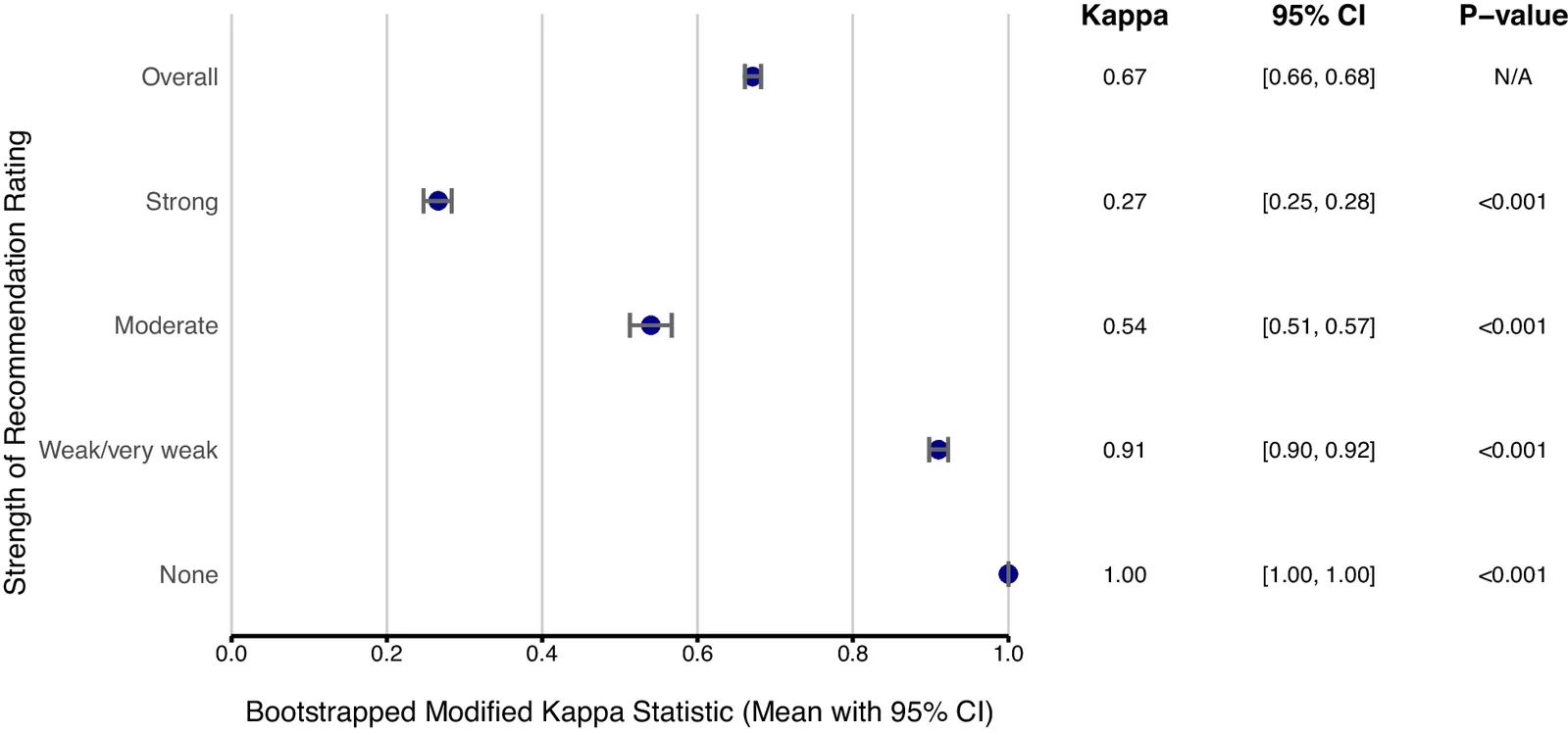
Concordance between strength of recommendation and level of evidence ratings overall and stratified by strength of recommendation ratings. *Note*: *P*-values cited in the manuscript reflect the average *P*-value for all individual pairwise comparison. For example, *P*-value < 0.001 for “Strong” recommendations compared to other subgroups reflects the average *P*-value for individual pairwise comparisons of “Strong” recommendations vs. other subgroups based on SOR rating (i.e., “Strong” vs. “Moderate”, “Strong” vs. “Weak/very weak”, “Strong” vs. “None”).

Overall, there was moderate concordance between SOR and LOE ratings (*κ* = 0.67, 95% CI 0.66–0.68) (Fig. 2). Concordance between SOR and LOE ratings varied significantly (*p* < 0.001) between strata of SOR rating. SOR-LOE rating concordance was minimal for strong recommendations (*κ* = 0.27, 95% CI 0.25–0.28), weak for moderate strength recommendations (*κ* = 0.54, 95% CI 0.51–0.56), and almost perfect for weak recommendations (*κ* = 0.91, 95% CI 0.90–0.92).

In our sensitivity analysis excluding recommendations with an SOR rating of “none,” recommendations with explicitly assigned strengths (i.e., strong, moderate, or weak) had weak SOR-LOE rating concordance (*κ* = 0.56, 95% CI 0.55–0.57).

### Subgroup Analyses

**Figure 3:**
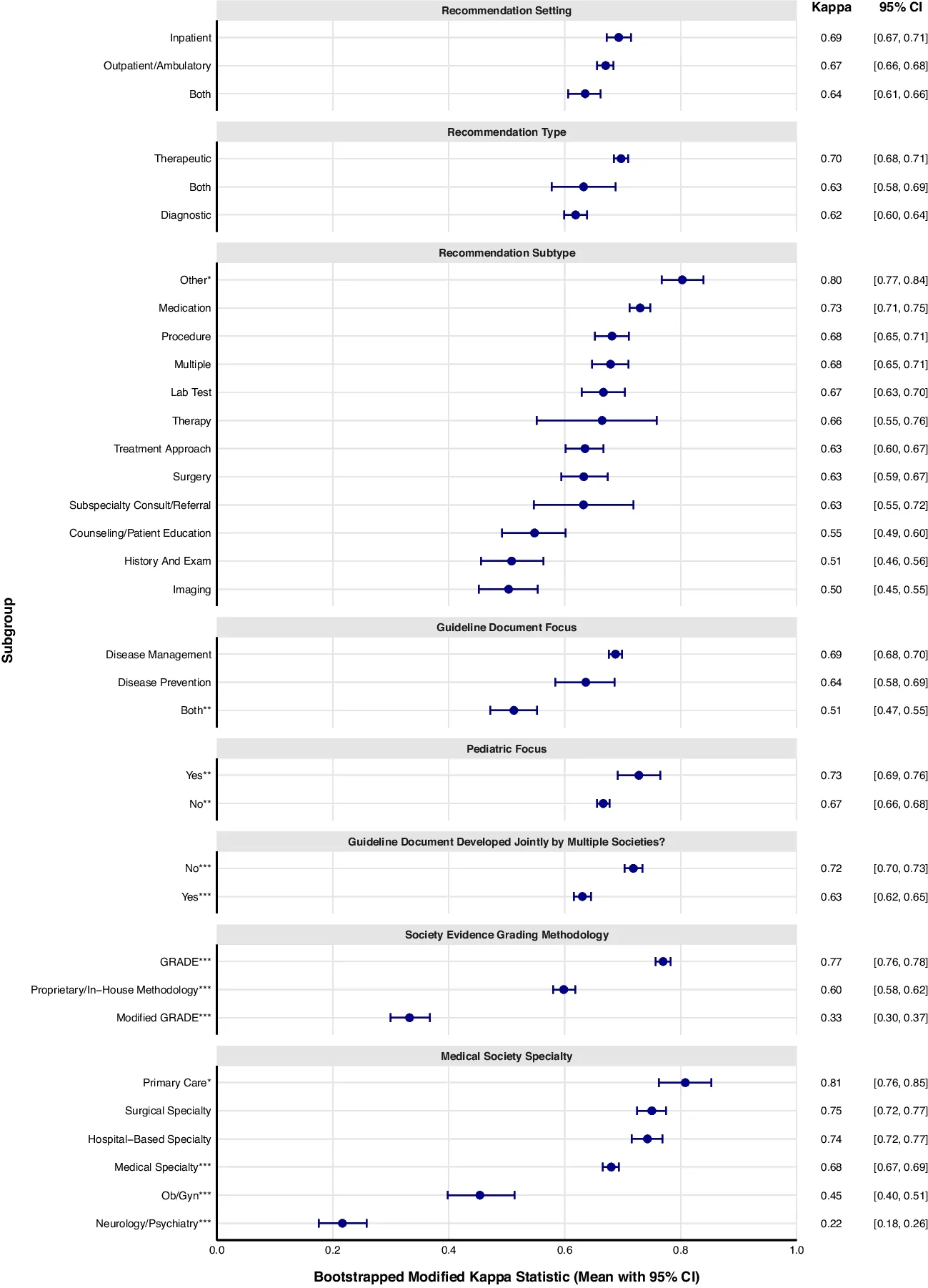
Concordance between strength of recommendation and level of evidence ratings by recommendation subgroups. A single asterisk indicates variables for which all other pairwise comparisons within each respective subgroup had a *P*-value of < 0.05 after Bonferroni correction for multiple comparisons. A double asterisk indicates variables for which all other pairwise comparisons within each respective subgroup had a *P*-value of < 0.01 after Bonferroni correction for multiple comparisons. A triple asterisk indicates variables for which all other pairwise comparisons within each respective subgroup had a *P*-value of < 0.001 after Bonferroni correction for multiple comparisons.

In Fig. 3, we present subgroup analyses where recommendations were categorized by recommendation-level, guideline-level, and society-level variables (also Supplement Tables 3 and 4). Regarding recommendation type, therapeutic recommendation had significantly greater (*p* < 0.001) SOR-LOE rating concordance (*κ* = 0.70, 95% CI 0.69–0.71) than diagnostic recommendations (*κ* = 0.62, 95% CI 0.60–0.64). Regarding guideline document-level variables, pediatrics-focused recommendations (*κ* = 0.73, 95% CI 0.69–0.76; *p* = 0.002) and recommendations from guidelines developed by a single society (*κ* = 0.72, 95% CI 0.70–0.73; *p* < 0.001) had statistically greater SOR-LOE rating concordance than their comparators.

SOR-LOE rating concordance was moderate for recommendations developed using the GRADE framework (*κ* = 0.77, 95% CI 0.76–0.78), moderate for recommendations developed using proprietary/in-house grading methodologies (*κ* = 0.60, 95% CI 0.58–0.62), and minimal for recommendations developed using modified GRADE frameworks (*κ* = 0.33, 95% CI 0.30–0.37). SOR-LOE rating concordance between all these methods was statistically different (p<0.001). Finally, recommendations developed by primary care (*p*  = 0.81, 95% CI 0.76–0.85), surgical (*κ* = 0.75, 95% CI 0.72–0.77), hospital-based (*κ* = 0.74, 95% CI 0.72–0.77), and medical specialty (*κ* = 0.68, 95% CI 0.67–0.69) societies had significantly greater SOR-LOE concordance (*p* < 0.001) than recommendations developed by obstetrics/gynecology (*κ* = 0.46, 95% CI 0.40–0.51) or neurology/psychiatry (*κ* = 0.22, 95% CI 0.18–0.26) specialty societies.

## DISCUSSION

In this cross-sectional analysis of 7582 recommendations within CPG documents issued by a multi-specialty cohort of US-based medical societies from 2019 to 2023, concordance between SOR and LOE ratings was moderate overall, but minimal for strongly rated recommendations. Moreover, one-third of all recommendations had LOE ratings weaker than their assigned SOR ratings, including three-quarters of recommendations with a strong SOR rating. Despite the IOM’s 2011 call^1^ to better align SOR and LOE ratings for CPG recommendations, our findings suggest room for further improvement, especially for recommendations with strong SOR ratings.

Our findings are consistent with prior research investigating recommendations issued by individual, US-based medical societies.^8–11^ Previous studies examining CPGs issued by the American Academy of Dermatology, Infectious Diseases Society of America, American College of Cardiology, and American College of Orthopaedic Surgeons raised concerns that, on average, less than one-third of recommendations were supported by strong evidence and that almost half of recommendations had discordant SOR and LOE ratings.^8–11^ Our study demonstrates that these phenomena broadly occur across a more diverse set of specialties and a larger sample of recently issued recommendations.

There are important reasons why discordance between SOR and LOE ratings may exist. The GRADE framework identifies five “paradigmatic situations” in which strong recommendations may be justified despite lower-quality evidence. These paradigmatic situations include instances where lower-quality evidence suggests that an intervention may be associated with (1) benefit in life-or-death clinical situations which are difficult to study systematically due to practical limitations, (2) high risk of patient harm with uncertain benefits, (3) potential equivalence to an alternative intervention where the alternative intervention is clearly less risky or costly, (4) similar benefits to an alternative intervention with the studied intervention having more associated risks or costs, and (5) potential catastrophic harm.^5,7^ GRADE framework developers emphasize, however, that making strong recommendations in the absence of strong evidence is generally discouraged.^7^ Given that most strong recommendations did not have strong evidence, societies may consider issuing such recommendations with weaker SOR ratings that more closely align with the quality of available evidence and that avoid overstating the certainty, consistency, and magnitude with which clinical benefits outweigh harms for clinical questions.

Discordance between SOR and LOE ratings may also exist because evidence-grading frameworks allow CPG document authors to upgrade or downgrade LOE ratings when evidence only indirectly justifies recommendations.^22^ For example, the American College of Cardiology’s 2022 heart failure guidelines strongly recommend medication-based blood pressure control for heart failure with preserved ejection fraction (HFpEF) patients, despite a lack of evidence pertaining to optimal blood pressure goals or antihypertensive regimens specifically for HFpEF.^23^ Accordingly, the recommendation’s LOE rating is weak, since the recommendation’s evidence is extrapolated from studies evaluating the treatment of general hypertension.^23^ For clinicians’ benefit, CPG authors may believe it is important to make strong recommendations despite weaker quality evidence if they believe there is unequivocally net clinical benefit.

Beyond variation in evidence grading frameworks and the downgrading of LOE for indirect evidence during evidence-to-decision determinations, several other practical limitations likely contribute to discordant SOR-LOE ratings. First, generating high-quality evidence can be infeasible or ethically complex for specific interventions or clinical scenarios, such as surgical procedures, emergency care, or rare diseases.^24–31^ Accordingly, CPG authors may rely on observational data when actionable guidance is needed despite a lack of available high-quality evidence. Second, diagnostic and preventive recommendations can be informed by studies utilizing surrogate markers or intermediate outcomes, such as diagnostic test accuracy or changes in patient biomarkers, which may not directly capture patient-centered endpoints (e.g., patient mortality) and therefore systematically lower formal LOE based on evidence-grading frameworks.^32–35^ Third, in diseases where research is rapidly evolving or disease prevalence is low, timely guidance from CPG authors may precede the generation of high-quality evidence.^29,31^ Finally, when empirical evidence is sparse, CPG authors may rely on expert consensus or existing standards of care to promote consistency in clinical practice, which is corroborated both by historical analyses^36,37^ and our observation that 36% of recommendations in our sample were informed by evidence with no LOE, which includes evidence based on expert consensus. These constraints are particularly salient in pediatrics, where ethical and logistical barriers to trials frequently necessitate extrapolation from non-pediatric settings or consensus-based recommendations.^38–41^

Despite these possible explanations for SOR-LOE rating discordance, our review of guideline texts suggested that, at times, recommendations lacked clear, upfront explanations for SOR-LOE rating discordance. Although we did not systematically quantify the presence or absence of such explanations, these impressions are consonant with prior IOM and GRADE developers’ concerns. We believe that when a recommendation’s SOR rating does not intuitively agree with its LOE rating, CPG authors should offer transparent explanations for their decision-making to bolster clinicians’ informed use of the recommendation in patient care. The American Academy of Pediatrics’ key action statements and College of American Pathologists’ appendix tables summarizing evidence-to-recommendation decisions are exemplary in this regard. Even just including brief narrative descriptions of how guideline writers determined certain SOR ratings despite relatively weaker LOE ratings would be informative. Improved transparency would not only avoid potentially misleading CPG end-users but also militate against claims that SOR-LOE rating discordance may be driven by external factors, such as CPG authors’ financial conflicts of interest.^42,43^ Future work should systematically characterize how often and how clearly guideline developers document distinct explanations for discordant SOR and LOE ratings in CPG document recommendations.

Alternatively, societies may consider adding qualifying statements to their upfront recommendation texts, such as “in the absence of reliable evidence, we recommend…” or “current evidence is insufficient to strongly recommend…,” when there is a relative lack of evidence for particular recommendations. Within our sample, the American Academy of Orthopaedic Surgeons and American Society of Anesthesiologists often included such qualifying statements alongside upfront recommendation texts. Finally, societies may consider approaching CPG documents as “living” documents, frequently revising recommendations’ SOR and LOE ratings as newer evidence necessitates; living guidelines were successfully iterated to guide COVID-19 care amidst a rapidly evolving evidence base.^44^ Future work could explore how clinicians interpret and are influenced by such evidence-to-recommendation rationales and qualifying statements.

Notably, the method medical societies use to appraise the quality of evidence and assign the strength of recommendations appears to matter. We found statistically greater SOR-LOE rating concordance for recommendations issued by societies that employ the GRADE framework compared with recommendations issued by societies using a modified GRADE rubric or a proprietary methodology. Societies that do not use GRADE cite concerns regarding GRADE’s poor internal consistency and low reliability,^45^ and challenges in using GRADE when there is a relative paucity of high-quality scientific evidence.^46^ This may be the case for certain clinical topics or medical specialties due to research funding priorities or the ethical challenges of conducting clinical trials in potentially vulnerable patient populations. To wit, recommendations by obstetrics/gynecology,^47,48^ neurology,^49,50^ and psychiatry^51,52^ societies had statistically poorer SOR-LOE concordance, with only the American Psychiatric Association using the GRADE framework among societies representing these specialties in our sample. Our finding that recommendations from pediatrics-focused CPGs had greater SOR-LOE rating concordance compared with non-pediatric CPGs despite a relative dearth of pediatrics-related clinical research^39−41^ and despite the American Academy of Pediatrics’ use of a proprietary evidence-grading methodology suggests that transparency, rigor, and circumspection in assigning SOR ratings in the face of limited evidence are achievable.

Our study has limitations. First, our definition of SOR-LOE rating concordance assumes that LOE should be the primary factor in determining SOR, while real-world evidence-to-recommendation decisions are likely multi-factorial. We did not systematically examine whether recommendations with SOR-LOE rating discordance provided plain-language justifications for discordant ratings, although capturing this nuance is challenging given variable and inconsistent reporting of guideline writers’ deliberations.^5,53^ Our study utilizes a conservative approach when assessing concordance, since we categorized all recommendations without an SOR rating as having concordant SOR-LOE ratings. In our sensitivity analyses excluding recommendations without SOR ratings, overall SOR-LOE rating concordance was weak, whereas concordance for the overall cohort of recommendations was moderate.^21^ Third, our findings may not generalize to non-US-based medical societies or US-based societies not included in our cohort.

Developing CPGs that effectively support clinicians in practicing evidence-based clinical decision-making and improving patients’ care and outcomes requires medical societies to be meticulous. Our findings underscore the need for continued scrutiny of the rigor and consistency of the methods medical societies’ CPG authors use to formulate recommendations so that assigned recommendation strengths appropriately reflect the quality of relevant evidence.

## Supplementary Information

Below is the link to the electronic supplementary material.

Supplementary Material 1 (DOCX 224 KB)

Supplementary Material 2 (PDF 190 KB)

## References

[1] Institute of Medicine Committee on Standards for Developing Trustworthy Clinical Practice Guidelines. Clinical practice guidelines we can trust. 2011.

[2] **Guerra-Farfan E, Garcia-Sanchez Y, Jornet-Gibert M, Nuñez JH, Balaguer-Castro M, Madden K.** Clinical practice guidelines: The good, the bad, and the ugly. Injury. 2023;54 Suppl 3:S26-S29. 10.1016/j.injury.2022.01.047. 35135686

[3] **Zimerman AL.** Evidence-based medicine: a short history of a modern medical movement. Virtual Mentor. 2013;15(1):71–6. 10.1001/virtualmentor.2013.15.1.mhst1-1301. 23356811

[4] **Thoma A, Eaves FF.** A brief history of evidence-based medicine (EBM) and the contributions of Dr David Sackett. Aesthet Surg J. 2015;35(8):NP261–3. 10.1093/asj/sjv130. 26163313

[5] **Neumann I, Santesso N, Akl EA, et al.** A guide for health professionals to interpret and use recommendations in guidelines developed with the GRADE approach. J Clin Epidemiol. 2016;72:45-55. 10.1016/j.jclinepi.2015.11.017. 26772609

[6] **LeFevre M.** From authority- to evidence-based medicine: are clinical practice guidelines moving us forward or backward? Ann Fam Med. 2017;15(5):410-12. 10.1370/afm.2141. PMC5593722 28893809

[7] **Andrews CJ, Schünemann JH, Oxman DA, et al.** GRADE guidelines: 15. Going from evidence to recommendation—determinants of a recommendation's direction and strength. J Clin Epidemiol. 2013;66(7):726–35. 10.1016/j.jclinepi.2013.02.003. 23570745

[8] **Cook C, Ottwell R, Rogers T, Checketts J, Musuvathy S, Vassar M.** Evaluation of the Level of Evidence Supporting the Recommendations Constituting the American Academy of Dermatology Clinical Practice Guidelines: Cross-Sectional Analysis. JMIR Dermatology. 2020;3(1):e17370. 10.2196/17370.

[9] **Miles EK, Rodriguez R, Gross EA, Kalil CA.** Strength of recommendation and quality of evidence for recommendations in current infectious diseases society of america guidelines. Open forum infectious diseases. 1/21/2021 2021;8(2) 10.1093/ofid/ofab033. PMC7885855 33614818

[10] **Bevan HG, Kalra A, Josephson AR, Al-Kindi GS.** Level of Scientific Evidence Underlying the Current American College of Cardiology/American Heart Association Clinical Practice Guidelines. Circ Cardiovasc Qual Outcomes. 2019;12(2). 10.1161/CIRCOUTCOMES.118.005293. 30755028

[11] **Reddy KA, Scott J, Checketts XJ, et al.** Levels of evidence backing the AAOS clinical practice guidelines. J Orthop Trauma Rehabil. 2021:221049172199253. 10.1177/2210491721992533.

[12] **Qumseya B, Goddard A, Qumseya A, Estores D, Draganov VP, Forsmark C.** Barriers to Clinical Practice Guideline Implementation Among Physicians: A Physician Survey. Int J Gen Med. 2021;14:7591-7598. 10.2147/IJGM.S333501. PMC8572046 34754231

[13] **Elm VE, Altman GD, Egger M, Pocock JS, Gøtzsche CP, Vandenbroucke PJ.** The Strengthening the Reporting of Observational Studies in Epidemiology (STROBE) statement: guidelines for reporting observational studies. PLoS Med. 2007;4(10):e296. 10.1371/journal.pmed.0040296. PMC2020495 17941714

[14] **Schwartz JA, Pearson SD.** Cost consideration in the clinical guidance documents of physician specialty societies in the United States. JAMA Intern Med. 2013;173(12):1091-7. 10.1001/jamainternmed.2013.817. 23649494

[15] Colleges AoAM (n.d.) U.S. Physician Workforce Data Dashboard. AAMC.org2024.

[16] ECRI. About ECRI Guidelines Trust ® ECRI Guidelines Trust 2024.

[17] **Sender JS.** ECRI INSTITUTE GUIDELINES TRUST. Journal of the Medical Library Association: JMLA; 2019.

[18] **Sim J, Wright CC.** The kappa statistic in reliability studies: use, interpretation, and sample size requirements. Phys Ther. 2005;85(3):257-68. 15733050

[19] **McHugh M.** Interrater reliability: the kappa statistic. Biochem Med (Zagreb). 2012;22(3):276–82. PMC3900052 23092060

[20] **Sedgwick P.** Multiple hypothesis testing and Bonferroni's correction. BMJ. 2014;349:g6284. 10.1136/bmj.g6284. 25331533

[21] **Viera AJ, Garrett JM.** Understanding interobserver agreement: the kappa statistic. Fam Med. 2005;37(5):360-3. 15883903

[22] **Guyatt G, Oxman DA, Akl AE, et al.** GRADE guidelines: 1. Introduction—GRADE evidence profiles and summary of findings tables. J Clin Epidemiol. 2011;64(4):383–394. 10.1016/j.jclinepi.2010.04.026. 21195583

[23] **Heidenreich AP, Bozkurt B, Aguilar D, et al.** 2022 AHA/ACC/HFSA Guideline for the Management of Heart Failure: A Report of the American College of Cardiology/American Heart Association Joint Committee on Clinical Practice Guidelines. Circulation. 2022;145(18) 10.1161/CIR.0000000000001063. 35363499

[24] **Mcculloch P, Cook AJ, Altman GD, Heneghan C, Diener KM.** IDEAL framework for surgical innovation 1: the idea and development stages. BMJ. 2013;346(jun18 3):f3012-f3012. 10.1136/bmj.f3012. PMC3685515 23778427

[25] **Ergina LP, Barkun SJ, Mcculloch P, Cook AJ, Altman GD.** IDEAL framework for surgical innovation 2: observational studies in the exploration and assessment stages. BMJ. 2013;346(jun18 3):f3011-f3011. 10.1136/bmj.f3011. PMC3685514 23778426

[26] **Cook AJ, Mcculloch P, Blazeby MJ, Beard JD, Marinac-Dabic D, Sedrakyan A.** IDEAL framework for surgical innovation 3: randomised controlled trials in the assessment stage and evaluations in the long term study stage. BMJ. 2013;346(jun18 3):f2820-20. 10.1136/bmj.f2820. PMC3685513 23778425

[27] **Vaslef NS.** Ethical and regulatory challenges associated with the exception from informed consent requirements for emergency research. Arch Surg. 2006;141(10):1019. 10.1001/archsurg.141.10.1019. 17043281

[28] **Morrison AC, Horwitz BI, Carrick MM.** Ethical and legal issues in emergency research: barriers to conducting prospective randomized trials in an emergency setting. J Surg Res. 2009;157(1):115-122. 10.1016/j.jss.2009.03.051. 19765724

[29] **Augustine FE, Adams RH, Mink WJ. **Clinical trials in rare disease. J Child Neurol. 2013;28(9):1142–50. 10.1177/0883073813495959. PMC3964003 24014509

[30] **Liu J, Barrett SJ, Leonardi TE, et al.** Natural History and Real‐World Data in Rare Diseases: Applications, Limitations, and Future Perspectives. J Clin Pharmacol. 2022;62(S2). 10.1002/jcph.2134. PMC10107901 36461748

[31] **Hilgers R-D, Heussen N.** Methodological approaches for generating robust evidence from trials in rare diseases. Z Evid Fortbild Qual Gesundhwes. 2024;189:66-72. 10.1016/j.zefq.2024.07.006.

[32] **Schünemann JH, Mustafa AR, Brozek J, et al.** GRADE guidelines: 22. The GRADE approach for tests and strategies—from test accuracy to patient-important outcomes and recommendations. J Clin Epidemiol. 2019;111:69–82. 10.1016/j.jclinepi.2019.02.003. 30738926

[33] **Schünemann JH, Oxman DA, Brozek J, et al.** Grading quality of evidence and strength of recommendations for diagnostic tests and strategies. BMJ. 2008;336(7653):1106-1110. 10.1136/bmj.39500.677199.AE. PMC2386626 18483053

[34] **Prasad V, Kim C, Burotto M, Vandross A.** The Strength of Association Between Surrogate End Points and Survival in Oncology. JAMA Intern Med. 2015;175(8):1389. 10.1001/jamainternmed.2015.2829. 26098871

[35] **Pasalic D, Mcginnis JG, Fuller DC, et al.** Progression-free survival is a suboptimal predictor for overall survival among metastatic solid tumour clinical trials. Eur J Cancer. 2020;136:176-85. 10.1016/j.ejca.2020.06.015. PMC7702022 32702645

[36] **Tricoci P.** Scientific Evidence Underlying the ACC/AHA Clinical Practice Guidelines. JAMA. 2009;301(8):831. 10.1001/jama.2009.205. 19244190

[37] **Fanaroff CA, Califf MR, Windecker S, Smith CS, Lopes DR.** Levels of Evidence Supporting American College of Cardiology/American Heart Association and European Society of Cardiology Guidelines, 2008-2018. JAMA. 2019;321(11):1069. 10.1001/jama.2019.1122. PMC6439920 30874755

[38] **Joseph DP, Craig CJ, Caldwell HYP.** Clinical trials in children. Br J Clin Pharmacol. 2015;79(3):357-369. 10.1111/bcp.12305. PMC4345947 24325152

[39] **Speer ME, Lee KL, Bourgeois TF, et al.** The state and future of pediatric research—an introductory overview. Pediatr Res. 2023; 10.1038/s41390-022-02439-4. PMC9873210 36694026

[40] **Fleurence LR, Forrest BC, Shuren J.** Strengthening the evidence base for pediatric medical devices using real-world data. J Pediatr. 2019;214:209-211. 10.1016/j.jpeds.2019.06.060. 31378521

[41] **Sampson RM, Benjamin KD, Cohen-Wolkowiez M.** Evidence-based guidelines for pediatric clinical trials: focus on StaR Child Health. Expert Rev Clin Pharmacol. 2012;5(5):525-531. 10.1586/ecp.12.52. PMC3566240 23121275

[42] **Mooghali M, Glick L, Ramachandran R, Ross JS.** Financial conflicts of interest among US physician authors of 2020 clinical practice guidelines: a cross-sectional study. BMJ Open. 2023;13(1):e069115. 10.1136/bmjopen-2022-069115. PMC9872463 36690402

[43] **Tabatabavakili S, Khan R, Scaffidi MA, Gimpaya N, Lightfoot D, Grover SC.** Financial Conflicts of Interest in Clinical Practice Guidelines: A Systematic Review. Mayo Clin Proc Innov Qual Outcomes. 2021;5(2):466-475. 10.1016/j.mayocpiqo.2020.09.016. PMC8105509 33997642

[44] **Agarwal A, Hunt JB, Stegemann M, et al.** A living WHO guideline on drugs for covid-19. BMJ. 2020:m3379. 10.1136/bmj.m3379. 32887691

[45] **Apfelbaum LJ, Connis TR.** The American Society of Anesthesiologists Practice Parameter Methodology. Anesthesiology. 2019;130(3):367–84. 10.1097/ALN.0000000000002551. 30724774

[46] **Butler JL, Danilack VA, O’Reilly NE, Zahn CM.** Clinical Practice Guideline Methodology. Obstet Gynecol. 2021;138(3):518-522. 10.1097/AOG.0000000000004519. 34412077

[47] Medicine Io. Women's Health Research: Progress, Pitfalls, Promise. The National Academies Press; 2010. 24983027

[48] **Gee ER, Wood FS, Schubert GK.** Women's Health, Pregnancy, and the U.S. Food and Drug Administration. Obstetrics Gynecol. 2014;123(1):161–165. 10.1097/AOG.0000000000000063. 24463677

[49] **Dorsey RE, Bloem RB.** The Parkinson pandemic—a call to action. JAMA Neurol. 2018;75(1):9. 10.1001/jamaneurol.2017.3299. 29131880

[50] **Feigin LV, Vos T.** Global burden of neurological disorders: from global burden of disease estimates to actions. Neuroepidemiology. 2019;52(1-2):1–2. 10.1159/000495197. 30472717

[51] **Patel V, Saxena S, Lund C, et al.** The Lancet Commission on global mental health and sustainable development. Lancet. 2018;392(10157):1553-98. 10.1016/S0140-6736(18)31612-X. 30314863

[52] **Collins YP, Patel V, Joestl SS, et al.** Grand challenges in global mental health. Nature. 2011;475(7354):27-30. 10.1038/475027a. PMC3173804 21734685

[53] ** Guyatt G, Rennie D, Meade MO, Cook DJ.** Users' guides to the medical literature: a manual for evidence-based clinical practice. 3rd ed. McGraw-Hill Education; 2015.

