# Supplementary material for: Level of Evidence and Strength of Recommendations in US Medical Society Clinical Practice Guidelines, 2019–2023: A Cross-Sectional Analysis
Source: J Gen Intern Med. 2025 Dec 22;41(12):3466–76. doi: 10.1007/s11606-025-10088-6 (PMC13569747; doi:10.1007/s11606-025-10088-6)
Supplement: Supplementary file 2 [file 11606_2025_10088_MOESM1_ESM.docx]

**SUPPLEMENTAL DOCUMENT**

**Level of Evidence and Strength of Recommendations in U.S. Medical Society Clinical Practice Guidelines, 2019-2023: A Cross-sectional Analysis**

**Authors:** Omar Qureshi, BS^1^; Joseph S. Ross, MD, MHS^2,3,4,5^; Reshma Ramachandran, MD, MPP, MHS^2,4,5^; Anand R. Habib, MD, MPhil, MHS^5,6^

**Affiliations:**

^1^Yale School of Medicine, New Haven, Connecticut

^2^Section of General Medicine, Department of Internal Medicine, Yale School of Medicine, New Haven, Connecticut

^3^Department of Health Policy and Management, Yale School of Public Health, New Haven, Connecticut

^4^Yale Collaboration for Regulatory Rigor, Integrity, and Transparency, Yale School of Medicine, New Haven, Connecticut

^5^National Clinician Scholars Program, Yale School of Medicine, New Haven, Connecticut

^6^Division of Hospital Medicine, University of California San Francisco, San Francisco, CA

**Contact information for corresponding author:** Anand R. Habib, MD, MPhil, MHS

**Mailing address:** University of California San Francisco School of Medicine; 521 Parnassus Ave, Box 0131, San Francisco, CA 94143

**Email:** anand.habib@ucsf.edu

**Telephone:** (415) 476-1000 (203) 785-2434

**TABLE OF CONTENTS**

**Supplement Tables**

- **Supplement Table 1:** Overview of Clinical Practice Guideline Document Recommendations Categorized by Subgroups Examined (Pages 3-5)
- **Supplement Table 2:** Summary of Clinical Practice Guideline Document Recommendations Grouped by Authoring Medical Society (Pages 6-8)
- **Supplement Table 3.** Bootstrapped Modified Kappa Statistic Estimates and 95% Confidence Intervals for Clinical Practice Guideline Document Recommendations Categorized by Subgroups (Page 9)
- **Supplement Table 4.** Pairwise Comparisons of Bootstrapped Modified Kappa Statistics and 95% Confidence Intervals with Bonferroni Correction for Clinical Practice Guideline Recommendations Categorized by Subgroup Analyzed (Pages 10-15)

**Supplement Figures**

- **Supplement Figure 1.** Inclusion and Exclusion Criteria Applied When Identifying and Selecting Clinical Practice Guideline (CPG) Documents for Analysis (Pages 16-17)

**Supplement Tables**

**Supplement Table 1. Overview of Clinical Practice Guideline Document Recommendations Categorized by Strength of Recommendation Rating, Level of Evidence Rating, and Subgroups Examined**

| **Overview of Clinical Practice Guideline Document Recommendations Categorized by Strength of Recommendation, Level of Evidence, and Subgroups Examined** | | | | | | | | | |
| --- | --- | --- | --- | --- | --- | --- | --- | --- | --- |
| **Data Subgroups** | **Strength of Recommendation** | | | | **Level of Evidence** | | | | **Total # of Recommendations (% of total)** |
|  | **Strong** | **Moderate** | **Weak** | **None** | **Strong** | **Moderate** | **Weak or Very Weak** | **None** |  |
| **Recommendation Setting** | | | | | | | | | |
| Inpatient | 598 (29%) | 265 (13%) | 589 (29%) | 577 (28%) | 128  (6%) | 404 (20%) | 620 (31%) | 877 (43%) | 2,029 (27%) |
| Outpatient/ Ambulatory | 1,348 (31%) | 870 (20%) | 1,088 (25%) | 1,069 (24%) | 529 (12%) | 1,029 (24%) | 1,340 (31%) | 1,477 (34%) | 4,375 (58%) |
| Both | 357 (30%) | 216 (18%) | 351 (30%) | 254 (22%) | 111  (9%) | 243 (21%) | 461 (39%) | 363 (31%) | 1,178 (16%) |
| **Recommendation Type** | | | | | | | | | |
| Diagnostic | 735 (32%) | 389 (17%) | 498 (22%) | 671 (29%) | 158  (7%) | 429 (19%) | 731 (32%) | 975 (43%) | 2,293 (30%) |
| Therapeutic | 1,482 (30%) | 925 (18%) | 1,457 (29%) | 1,153 (23%) | 590 (12%) | 1,211 (24%) | 1,597 (32%) | 1619 (32%) | 5,017 (66%) |
| Both | 86 (32%) | 37  (14%) | 73 (27%) | 76 (28%) | 20  (7%) | 36  (13%) | 93 (34%) | 123 (45%) | 272 (4%) |
| **Recommendation Subtype** | | | | | | | | | |
| Counseling/  Patient Education | 94 (31%) | 83  (28%) | 41 (14%) | 83 (28%) | 27  (9%) | 69  (23%) | 66 (22%) | 139 (46%) | 301 (4%) |
| History And Exam | 135 (42%) | 57  (18%) | 57 (18%) | 76 (23%) | 33  (10%) | 55  (17%) | 86 (26%) | 151 (46%) | 325 (4%) |
| Imaging | 134 (36%) | 110 (29%) | 70 (19%) | 62 (16%) | 15  (4%) | 117 (31%) | 120 (32%) | 124 (33%) | 376 (5%) |
| Lab Test | 176 (33%) | 85  (16%) | 155 (29%) | 121 (23%) | 56  (10%) | 106 (20%) | 219 (41%) | 156 (29%) | 537 (7%) |
| Medication | 727 (32%) | 337 (15%) | 798 (35%) | 445 (19%) | 338 (15%) | 563 (24%) | 835 (36%) | 571 (25%) | 2,307 (30%) |
| Procedure | 237 (28%) | 148 (17%) | 241 (28%) | 227 (27%) | 71  (8%) | 162 (19%) | 289 (34%) | 331 (39%) | 853 (11%) |
| Subspecialty Consult/Referral | 32 (25%) | 18  (14%) | 23 (18%) | 55 (43%) | 6  (5%) | 14  (11%) | 32 (25%) | 76 (59%) | 128 (2%) |
| Surgery | 151 (30%) | 141 (28%) | 127 (25%) | 91 (18%) | 55  (11%) | 149 (29%) | 156 (31%) | 150 (29%) | 510 (7%) |
| Therapy | 31 (36%) | 22  (25%) | 24 (28%) | 10 (11%) | 15  (17%) | 28  (32%) | 25 (29%) | 19 (22%) | 87 (1%) |
| Treatment Approach | 253 (31%) | 120 (15%) | 165 (20%) | 288 (35%) | 42  (5%) | 129 (16%) | 221 (27%) | 434 (53%) | 826 (11%) |
| Other | 88 (20%) | 58  (13%) | 103 (23%) | 193 (44%) | 34  (8%) | 72  (16%) | 114 (26%) | 222 (50%) | 442 (6%) |
| Multiple | 245 (28%) | 172 (19%) | 224 (25%) | 249 (28%) | 76  (9%) | 212 (24%) | 258 (29%) | 344 (39%) | 890 (12%) |
| **Guideline Document Focus** | | | | | | | | | |
| Disease Management | 1,945 (29%) | 1,180 (18%) | 1,777 (27%) | 1,727 (26%) | 681 (10%) | 1,489 (22%) | 2,144 (32%) | 2,315 (35%) | 6,629 (87%) |
| Disease Prevention | 134 (40%) | 51  (15%) | 112 (34%) | 37 (11%) | 44  (13%) | 97  (29%) | 104 (31%) | 89 (27%) | 334 (4%) |
| Both | 224 (36%) | 120 (19%) | 139 (22%) | 136 (22%) | 43  (7%) | 90  (15%) | 173 (28%) | 313 (51%) | 619 (8%) |
| **Pediatric Focus** | | | | | | | | | |
| Yes | 119 (21%) | 140 (25%) | 160 (29%) | 142 (25%) | 59  (11%) | 111  (20%) | 168 (30%) | 223 (40%) | 561 (7%) |
| No | 2,184 (31%) | 1,211 (17%) | 1,868 (27%) | 1,758 (25%) | 709 (10%) | 1,565 (22%) | 2253 (32%) | 2494 (36%) | 7,021 (93%) |
| **Guideline Document Developed Jointly by Multiple Societies?** | | | | | | | | | |
| Yes | 1,448 (35%) | 827 (20%) | 939 (23%) | 866 (21%) | 470 (12%) | 1,083 (27%) | 1,135 (28%) | 1,392 (34%) | 4,080 (54%) |
| No | 855 (24%) | 524 (15%) | 1,089 (31%) | 1,034 (30%) | 298  (9%) | 593 (17%) | 1,286 (37%) | 1,325 (38%) | 3,502 (46%) |
| **Society Evidence Grading Methodology** | | | | | | | | | |
| GRADE | 1,122  (26%) | 422  (10%) | 1,501  (35%) | 1,209  (29%) | 428 (10%) | 745 (18%) | 1639 (38%) | 1442 (34%) | 4,254 (56%) |
| Modified GRADE | 250  (37%) | 241  (36%) | 159  (24%) | 17  (3%) | 106 (16%) | 88  (13%) | 131 (20%) | 342 (51%) | 667 (9%) |
| Propriety/In-house methodology | 931  (35%) | 688  (26%) | 368  (14%) | 674  (25%) | 234  (9%) | 843 (32%) | 651 (24%) | 933 (35%) | 2,661 (35%) |
| **Medical Society Specialty** | | | | | | | | | |
| Hospital-Based Specialty | 278 (26%) | 119  (11%) | 299 (28%) | 363 (34%) | 66  (6%) | 176 (17%) | 308 (29%) | 509 (48%) | 1,059 (14%) |
| Medical Specialty | 1,447 (32%) | 648 (14%) | 1,316 (29%) | 1,063 (24%) | 416  (9%) | 1,130 (25%) | 1,573 (35%) | 1,355 (30%) | 4,474 (59%) |
| Primary Care | 68 (24%) | 48  (17%) | 38 (13%) | 132 (46%) | 41  (14%) | 53  (19%) | 45 (16%) | 147 (51%) | 286 (4%) |
| Surgical Specialty | 234 (22%) | 295 (28%) | 206 (19%) | 325 (31%) | 138 (13%) | 218 (21%) | 340 (32%) | 364 (34%) | 1,060 (14%) |
| Neurology/ Psychiatry | 153  (42%) | 151  (41%) | 56  (15%) | 4  (1%) | 49  (%) | 30  (%) | 57  (%) | 228  (%) | 364  (5%) |
| Ob/Gyn | 123 (36%) | 90  (27%) | 113 (33%) | 13 (4%) | 58  (17%) | 69  (20%) | 98 (29%) | 114 (34%) | 339 (4%) |

**Supplement Table 2. Summary of Clinical Practice Guideline Document Recommendations Grouped by Authoring Medical Society**

| Authoring Medical Society | Strength of recommendation | | | | Level of evidence | | | |  |  |
| --- | --- | --- | --- | --- | --- | --- | --- | --- | --- | --- |
|  | Strong | Moderate | Weak/very weak | None | Strong | Moderate | Weak/very weak | None | Recommendations from Society | % of Total Recommendations |
| American Academy of Dermatology (AAD) | 103 | 134 | 107 | 92 | 157 | 108 | 78 | 93 | 436 | 5.8% |
| American Academy of Family Physicians (AAFP) | 2 | 0 | 2 | 0 | 1 | 2 | 1 | 0 | 4 | 0.1% |
| American Academy of Neurology (AAN) | 125 | 151 | 44 | 4 | 47 | 17 | 32 | 228 | 324 | 4.3% |
| American Academy of Orthopedic Surgeons (AAOS) | 77 | 80 | 96 | 47 | 94 | 66 | 97 | 43 | 300 | 4.0% |
| American Academy of Otolaryngology - Head and Neck Surgery Foundation (AAO HNS) | 17 | 65 | 11 | 14 | 10 | 34 | 49 | 14 | 107 | 1.4% |
| American Academy of Pediatrics (AAP) | 62 | 48 | 13 | 96 | 38 | 43 | 23 | 115 | 219 | 2.9% |
| American College of Cardiology (ACC) | 570 | 328 | 149 | 29 | 104 | 551 | 290 | 131 | 1076 | 14.2% |
| American College of Chest Physicians (ACCP) | 50 | 4 | 113 | 108 | 0 | 30 | 110 | 135 | 275 | 3.6% |
| American College of Emergency Physicians (ACEP) | 2 | 16 | 21 | 8 | 0 | 22 | 18 | 7 | 47 | 0.6% |
| American College of Gastroenterology (ACG) | 189 | 0 | 232 | 396 | 11 | 99 | 312 | 395 | 817 | 10.8% |
| American College of Obstetrics and Gynecology (ACOG) | 123 | 90 | 113 | 13 | 58 | 69 | 98 | 114 | 339 | 4.5% |
| American College of Physicians (ACP) | 4 | 0 | 23 | 36 | 2 | 8 | 21 | 32 | 63 | 0.8% |
| American Gastroenterological Association (AGA) | 22 | 0 | 97 | 174 | 4 | 49 | 73 | 167 | 293 | 3.9% |
| American Psychiatric Association (APA) | 28 | 0 | 12 | 0 | 2 | 13 | 25 | 0 | 40 | 0.5% |
| American Society of Anesthesiologists (ASA) | 140 | 81 | 75 | 263 | 48 | 75 | 77 | 359 | 559 | 7.4% |
| American Society of Clinical Oncology (ASCO) | 221 | 133 | 65 | 53 | 100 | 141 | 109 | 122 | 472 | 6.2% |
| American Society of Gastrointestinal Endoscopy (ASGE) | 32 | 0 | 63 | 12 | 3 | 24 | 68 | 12 | 107 | 1.4% |
| American Society of Hematology (ASH) | 51 | 5 | 236 | 37 | 5 | 43 | 231 | 50 | 329 | 4.3% |
| American Thoracic Society (ATS) | 71 | 8 | 149 | 26 | 7 | 37 | 158 | 52 | 254 | 3.4% |
| American Urological Association (AUA) | 140 | 150 | 99 | 264 | 34 | 118 | 194 | 307 | 653 | 8.6% |
| College of American Pathologists (CAP) | 47 | 22 | 17 | 26 | 11 | 24 | 33 | 44 | 112 | 1.5% |
| Society of Critical Care Medicine (SCCM) | 89 | 0 | 186 | 66 | 7 | 55 | 180 | 99 | 341 | 4.5% |
| The Endocrine Society (TES) | 138 | 36 | 105 | 136 | 25 | 48 | 144 | 198 | 415 | 5.5% |
| Total | **2303** | **1351** | **2028** | **1900** | **768** | **1676** | **2421** | **2717** | **7582** | **100.0%** |

**Supplement Table 3. Bootstrapped Modified Kappa Statistic Estimates and 95% Confidence Intervals for Clinical Practice Guideline Document Recommendations Categorized by Subgroups**

| **Subgroup** | **Bootstrapped Modified Kappa Estimate** | **Lower Bound of 95% Confidence Interval** | **Upper Bound of 95% Confidence Interval** |
| --- | --- | --- | --- |
| **Recommendation Setting** | | | |
| Outpatient/Ambulatory | 0.67 | 0.66 | 0.68 |
| Inpatient | 0.69 | 0.67 | 0.71 |
| Both | 0.63 | 0.61 | 0.66 |
| **Recommendation Type** | | | |
| Diagnostic | 0.62 | 0.60 | 0.64 |
| Therapeutic | 0.70 | 0.69 | 0.71 |
| Both | 0.63 | 0.57 | 0.68 |
| **Recommendation Subtype** | | | |
| Procedure | 0.68 | 0.65 | 0.71 |
| History And Exam | 0.51 | 0.46 | 0.56 |
| Other | 0.80 | 0.76 | 0.84 |
| Lab Test | 0.67 | 0.63 | 0.71 |
| Surgery | 0.63 | 0.59 | 0.67 |
| Counseling/Patient Education | 0.55 | 0.50 | 0.60 |
| Multiple | 0.68 | 0.65 | 0.71 |
| Imaging | 0.50 | 0.45 | 0.55 |
| Treatment Approach | 0.63 | 0.60 | 0.67 |
| Medication | 0.73 | 0.71 | 0.75 |
| Subspecialty Consult/Referral | 0.63 | 0.55 | 0.72 |
| Therapy | 0.67 | 0.56 | 0.76 |
| **Guideline Document Focus** | | | |
| Disease Management | 0.69 | 0.68 | 0.70 |
| Disease Prevention | 0.64 | 0.59 | 0.69 |
| Both | 0.51 | 0.47 | 0.55 |
| **Pediatric Focus** | | | |
| Yes | 0.73 | 0.69 | 0.76 |
| No | 0.67 | 0.66 | 0.68 |
| **Guideline Document Developed Jointly by Multiple Societies** | | | |
| Yes | 0.63 | 0.61 | 0.65 |
| No | 0.72 | 0.70 | 0.73 |
| **Society Evidence Grading Methodology** | | | |
| GRADE | 0.77 | 0.76 | 0.78 |
| Proprietary/In-House Methodology | 0.60 | 0.58 | 0.62 |
| Modified GRADE Framework | 0.33 | 0.30 | 0.37 |
| **Medical Society Specialty** | | | |
| Medical Specialty | 0.68 | 0.67 | 0.69 |
| Primary Care | 0.81 | 0.76 | 0.85 |
| Neurology/Psychiatry | 0.22 | 0.18 | 0.26 |
| Surgical Specialty | 0.75 | 0.72 | 0.77 |
| Hospital-Based Specialty | 0.74 | 0.72 | 0.77 |
| Obstetrics/Gynecology | 0.46 | 0.40 | 0.51 |

**Supplement Table 4. Pairwise Comparisons of Bootstrapped Modified Kappa Statistics and 95% Confidence Intervals with Bonferroni Correction for Clinical Practice Guideline Recommendations Categorized by Subgroup Analyzed**

| **Pairwise Comparison** | **Difference in Modified Kappa statistic** | **Lower Bound of 95% CI** | **Upper Bound of 95% CI** | **P-value** | **Difference significant before Bonferroni correction?*** | **Bonferroni correction adjusted P-Value threshold*^†^*** | **Difference significant after Bonferroni correction? *^†^*** |
| --- | --- | --- | --- | --- | --- | --- | --- |
| **Recommendation Setting** | | | | | | | |
| Inpatient vs. Outpatient/Ambulatory | 0.022 | -0.003 | 0.046 | 0.0740 | No | 0.0167 | No |
| Inpatient vs. Both | 0.058 | 0.024 | 0.093 | <0.001 | Yes | 0.0167 | Yes |
| Outpatient/Ambulatory vs. Both | 0.036 | 0.006 | 0.065 | 0.0200 | Yes | 0.0167 | No |
| **Recommendation Type** | | | | | | | |
| Diagnostic vs. Therapeutic | -0.078 | -0.102 | -0.056 | <0.001 | Yes | 0.0167 | Yes |
| Diagnostic vs. Both | -0.014 | -0.075 | 0.048 | 0.6320 | No | 0.0167 | No |
| Therapeutic vs. Both | 0.065 | 0.005 | 0.124 | 0.0360 | Yes | 0.0167 | No |
| **Recommendation Subtype** | | | | | | | |
| Counseling/Patient Education vs. History and Exam | 0.037 | -0.048 | 0.122 | 0.3520 | No | 0.0008 | No |
| Counseling/Patient Education vs. Imaging | 0.046 | -0.027 | 0.128 | 0.2260 | No | 0.0008 | No |
| Counseling/Patient Education vs. Lab Test | -0.118 | -0.194 | -0.048 | <0.001 | Yes | 0.0008 | Yes |
| Counseling/Patient Education vs. Medication | -0.182 | -0.239 | -0.125 | <0.001 | Yes | 0.0008 | Yes |
| Counseling/Patient Education vs. Multiple | -0.130 | -0.194 | -0.068 | <0.001 | Yes | 0.0008 | Yes |
| Counseling/Patient Education vs. Other | -0.255 | -0.324 | -0.188 | <0.001 | Yes | 0.0008 | Yes |
| Counseling/Patient Education vs. Procedure | -0.133 | -0.193 | -0.070 | <0.001 | Yes | 0.0008 | Yes |
| Counseling/Patient Education vs. Subspecialty Consult/Referral | -0.085 | -0.185 | 0.024 | 0.1320 | No | 0.0008 | No |
| Counseling/Patient Education vs. Surgery | -0.085 | -0.155 | -0.018 | 0.0140 | Yes | 0.0008 | No |
| Counseling/Patient Education vs. Therapy | -0.118 | -0.237 | -0.007 | 0.0400 | Yes | 0.0008 | No |
| Counseling/Patient Education vs. Treatment Approach | -0.086 | -0.150 | -0.020 | 0.0140 | Yes | 0.0008 | No |
| History and Exam vs. Imaging | 0.008 | -0.060 | 0.084 | 0.8120 | No | 0.0008 | No |
| History and Exam vs. Lab Test | -0.156 | -0.222 | -0.090 | <0.001 | Yes | 0.0008 | Yes |
| History and Exam vs. Medication | -0.219 | -0.277 | -0.164 | <0.001 | Yes | 0.0008 | Yes |
| History and Exam vs. Multiple | -0.168 | -0.234 | -0.111 | <0.001 | Yes | 0.0008 | Yes |
| History and Exam vs. Other | -0.292 | -0.357 | -0.223 | <0.001 | Yes | 0.0008 | Yes |
| History and Exam vs. Procedure | -0.170 | -0.228 | -0.110 | <0.001 | Yes | 0.0008 | Yes |
| History and Exam vs. Subspecialty Consult/Referral | -0.122 | -0.219 | -0.030 | 0.0220 | Yes | 0.0008 | No |
| History and Exam vs. Surgery | -0.123 | -0.189 | -0.055 | 0.0020 | Yes | 0.0008 | No |
| History and Exam vs. Therapy | -0.156 | -0.271 | -0.039 | 0.0060 | Yes | 0.0008 | No |
| History and Exam vs. Treatment Approach | -0.124 | -0.192 | -0.060 | <0.001 | Yes | 0.0008 | Yes |
| Imaging vs. Lab Test | -0.164 | -0.227 | -0.098 | <0.001 | Yes | 0.0008 | Yes |
| Imaging vs. Medication | -0.227 | -0.282 | -0.170 | <0.001 | Yes | 0.0008 | Yes |
| Imaging vs. Multiple | -0.176 | -0.233 | -0.118 | <0.001 | Yes | 0.0008 | Yes |
| Imaging vs. Other | -0.301 | -0.361 | -0.240 | <0.001 | Yes | 0.0008 | Yes |
| Imaging vs. Procedure | -0.178 | -0.237 | -0.120 | <0.001 | Yes | 0.0008 | Yes |
| Imaging vs. Subspecialty Consult/Referral | -0.130 | -0.224 | -0.035 | 0.0040 | Yes | 0.0008 | No |
| Imaging vs. Surgery | -0.131 | -0.195 | -0.064 | <0.001 | Yes | 0.0008 | Yes |
| Imaging vs. Therapy | -0.164 | -0.272 | -0.053 | 0.0040 | Yes | 0.0008 | No |
| Imaging vs. Treatment Approach | -0.132 | -0.191 | -0.075 | <0.001 | Yes | 0.0008 | Yes |
| Lab Test vs. Medication | -0.063 | -0.107 | -0.022 | 0.0020 | Yes | 0.0008 | No |
| Lab Test vs. Multiple | -0.012 | -0.063 | 0.039 | 0.6660 | No | 0.0008 | No |
| Lab Test vs. Other | -0.137 | -0.195 | -0.085 | <0.001 | Yes | 0.0008 | Yes |
| Lab Test vs. Procedure | -0.014 | -0.064 | 0.033 | 0.5640 | No | 0.0008 | No |
| Lab Test vs. Subspecialty Consult/Referral | 0.034 | -0.050 | 0.124 | 0.4440 | No | 0.0008 | No |
| Lab Test vs. Surgery | 0.033 | -0.027 | 0.093 | 0.2780 | No | 0.0008 | No |
| Lab Test vs. Therapy | 0.000 | -0.103 | 0.103 | 0.9900 | No | 0.0008 | No |
| Lab Test vs. Treatment Approach | 0.032 | -0.019 | 0.089 | 0.2100 | No | 0.0008 | No |
| Medication vs. Multiple | 0.051 | 0.016 | 0.088 | 0.0080 | Yes | 0.0008 | No |
| Medication vs. Other | -0.073 | -0.114 | -0.032 | <0.001 | Yes | 0.0008 | Yes |
| Medication vs. Procedure | 0.049 | 0.014 | 0.084 | 0.0140 | Yes | 0.0008 | No |
| Medication vs. Subspecialty Consult/Referral | 0.097 | 0.013 | 0.180 | 0.0340 | Yes | 0.0008 | No |
| Medication vs. Surgery | 0.097 | 0.051 | 0.139 | <0.001 | Yes | 0.0008 | Yes |
| Medication vs. Therapy | 0.063 | -0.032 | 0.166 | 0.2040 | No | 0.0008 | No |
| Medication vs. Treatment Approach | 0.096 | 0.061 | 0.134 | <0.001 | Yes | 0.0008 | Yes |
| Multiple vs. Other | -0.125 | -0.171 | -0.079 | <0.001 | Yes | 0.0008 | Yes |
| Multiple vs. Procedure | -0.002 | -0.051 | 0.043 | 0.9260 | No | 0.0008 | No |
| Multiple vs. Subspecialty Consult/Referral | 0.046 | -0.040 | 0.139 | 0.2880 | No | 0.0008 | No |
| Multiple vs. Surgery | 0.045 | -0.007 | 0.099 | 0.0840 | No | 0.0008 | No |
| Multiple vs. Therapy | 0.012 | -0.088 | 0.121 | 0.7620 | No | 0.0008 | No |
| Multiple vs. Treatment Approach | 0.044 | -0.004 | 0.085 | 0.0680 | No | 0.0008 | No |
| Other vs. Procedure | 0.122 | 0.070 | 0.173 | <0.001 | Yes | 0.0008 | Yes |
| Other vs. Subspecialty Consult/Referral | 0.170 | 0.079 | 0.263 | <0.001 | Yes | 0.0008 | Yes |
| Other vs. Surgery | 0.170 | 0.117 | 0.226 | <0.001 | Yes | 0.0008 | Yes |
| Other vs. Therapy | 0.137 | 0.031 | 0.244 | 0.0120 | Yes | 0.0008 | No |
| Other vs. Treatment Approach | 0.169 | 0.117 | 0.218 | <0.001 | Yes | 0.0008 | Yes |
| Procedure vs. Subspecialty Consult/Referral | 0.048 | -0.036 | 0.142 | 0.2560 | No | 0.0008 | No |
| Procedure vs. Surgery | 0.048 | 0.000 | 0.098 | 0.0560 | No | 0.0008 | No |
| Procedure vs. Therapy | 0.014 | -0.089 | 0.118 | 0.7720 | No | 0.0008 | No |
| Procedure vs. Treatment Approach | 0.047 | 0.002 | 0.091 | 0.0440 | Yes | 0.0008 | No |
| Subspecialty Consult/Referral vs. Surgery | -0.001 | -0.089 | 0.095 | 0.9780 | No | 0.0008 | No |
| Subspecialty Consult/Referral vs. Therapy | -0.034 | -0.165 | 0.100 | 0.6180 | No | 0.0008 | No |
| Subspecialty Consult/Referral vs. Treatment Approach | -0.002 | -0.092 | 0.079 | 0.9040 | No | 0.0008 | No |
| Surgery vs. Therapy | -0.033 | -0.135 | 0.078 | 0.5180 | No | 0.0008 | No |
| Surgery vs. Treatment Approach | -0.001 | -0.052 | 0.053 | 0.9240 | No | 0.0008 | No |
| Therapy vs. Treatment Approach | 0.032 | -0.075 | 0.135 | 0.5240 | No | 0.0008 | No |
| **Guideline Document Focus** | | | | | | | |
| Disease Management vs. Disease Prevention | 0.050 | -0.002 | 0.104 | 0.0640 | No | 0.0167 | No |
| Disease Management vs. Both | 0.175 | 0.132 | 0.217 | <0.001 | Yes | 0.0167 | Yes |
| Disease Prevention vs. Both | 0.126 | 0.063 | 0.192 | 0.0020 | Yes | 0.0167 | Yes |
| **Pediatrics Focus** | | | | | | | |
| Yes vs. No | -0.061 | -0.096 | -0.023 | 0.0020 | Yes | 0.0500 | Yes |
| **Guideline Document Developed Jointly by Multiple Societies** | | | | | | | |
| Yes vs. No | 0.088 | 0.067 | 0.110 | <0.001 | Yes | 0.0500 | Yes |
| **Society Evidence Grading Methodology** | | | | | | | |
| GRADE vs. Modified GRADE | 0.437 | 0.398 | 0.473 | <0.001 | Yes | 0.0167 | Yes |
| GRADE vs. Proprietary/in-House Methodology | 0.172 | 0.149 | 0.195 | <0.001 | Yes | 0.0167 | Yes |
| Modified GRADE vs. Proprietary/In-House Methodology | -0.265 | -0.306 | -0.226 | <0.001 | Yes | 0.0167 | Yes |
| **Medical Society Specialty** | | | | | | | |
| Hospital-Based Specialty vs. Medical Specialty | 0.062 | 0.033 | 0.092 | <0.001 | Yes | 0.0033 | Yes |
| Hospital-Based Specialty vs. Neurology/Psychiatry | 0.525 | 0.476 | 0.575 | <0.001 | Yes | 0.0033 | Yes |
| Hospital-Based Specialty vs. Ob/Gyn | 0.288 | 0.233 | 0.343 | <0.001 | Yes | 0.0033 | Yes |
| Hospital-Based Specialty vs. Primary Care | -0.065 | -0.122 | -0.016 | 0.0060 | Yes | 0.0033 | No |
| Hospital-Based Specialty vs. Surgical Specialty | -0.008 | -0.045 | 0.028 | 0.6140 | No | 0.0033 | No |
| Medical Specialty vs. Neurology/Psychiatry | 0.463 | 0.419 | 0.509 | <0.001 | Yes | 0.0033 | Yes |
| Medical Specialty vs. Ob/Gyn | 0.226 | 0.169 | 0.278 | <0.001 | Yes | 0.0033 | Yes |
| Medical Specialty vs. Primary Care | -0.128 | -0.177 | -0.078 | <0.001 | Yes | 0.0033 | Yes |
| Medical Specialty vs. Surgical Specialty | -0.070 | -0.099 | -0.042 | <0.001 | Yes | 0.0033 | Yes |
| Neurology/Psychiatry vs. Ob/Gyn | -0.237 | -0.303 | -0.169 | <0.001 | Yes | 0.0033 | Yes |
| Neurology/Psychiatry vs. Primary Care | -0.591 | -0.653 | -0.525 | <0.001 | Yes | 0.0033 | Yes |
| Neurology/Psychiatry vs. Surgical Specialty | -0.533 | -0.579 | -0.484 | <0.001 | Yes | 0.0033 | Yes |
| Ob/Gyn vs. Primary Care | -0.353 | -0.423 | -0.282 | <0.001 | Yes | 0.0033 | Yes |
| Ob/Gyn vs. Surgical Specialty | -0.296 | -0.356 | -0.239 | <0.001 | Yes | 0.0033 | Yes |
| Primary Care vs. Surgical Specialty | 0.058 | 0.006 | 0.111 | 0.0260 | Yes | 0.0033 | No |

**A pairwise comparison was considered statistically significant before Bonferroni correction if the p-value of the comparison was <0.05, when the calculated difference in kappa statistic was non-zero, and when the 95% CI of the difference in kappa estimate was non-zero*

*† A pairwise comparison was considered statistically significant after Bonferroni correction if the P-value was less than the adjusted P-value threshold (0.05/number of pairwise comparisons), when the calculated difference in kappa statistic was non-zero, and when the 95% CI of the difference in kappa estimate was non-zero*

*Abbreviations: CI = confidence interval, Ob/gyn = Obstetrics and Gynecology*

**Supplemental Figures**

**Supplement Figure 1. Inclusion and Exclusion Criteria Applied When Identifying and Selecting Clinical Practice Guideline (CPG) Documents for Analysis**

*Abbreviations:*

*ECRI = Emergency Care Research Institute, AMA = American Medical Association, AOA = American Osteopathic Association, APICE = Associated for Professionals in Infection Control and Epidemiology, ACS = American College of Surgeons, ACSM = American College of Sports Medicine, SHM = Society of Hospital Medicine, AAOS = American Academy of Orthopaedic Surgeons, ACC = American College of Cardiology, ACR = American College of Radiology, ASA = American Society of Anesthesiologists , ACOG = American College of Obstetricians and Gynecologists, AGA = American Gastroenterological Society, ASCO = American Society of Clinical Oncology*
